# Predicting building types using OpenStreetMap

**DOI:** 10.1038/s41598-022-24263-w

**Published:** 2022-11-20

**Authors:** Kuldip Singh Atwal, Taylor Anderson, Dieter Pfoser, Andreas Züfle

**Affiliations:** 1 Geography and Geoinformation Science, George Mason University, Fairfax, VA 22030 USA; 2 Department of Computer Science, Emory University, Atlanta, GA 30322 USA

**Keywords:** Computer science, Data processing, Databases, Civil engineering, Sustainability

## Abstract

Having accurate building information is paramount for a plethora of applications, including humanitarian efforts, city planning, scientific studies, and navigation systems. While volunteered geographic information from sources such as OpenStreetMap (OSM) has good building geometry coverage, descriptive attributes such as the type of a building are sparse. To fill this gap, this study proposes a supervised learning-based approach to provide meaningful, semantic information for OSM data without manual intervention. We present a basic demonstration of our approach that classifies buildings into either *residential* or *non-residential* types for three study areas: Fairfax County in Virginia (VA), Mecklenburg County in North Carolina (NC), and the City of Boulder in Colorado (CO). The model leverages (i) available OSM tags capturing non-spatial attributes, (ii) geometric and topological properties of the building footprints including adjacent types of roads, proximity to parking lots, and building size. The model is trained and tested using ground truth data available for the three study areas. The results show that our approach achieves high accuracy in predicting building types for the selected areas. Additionally, a trained model is transferable with high accuracy to other regions where ground truth data is unavailable. The OSM and data science community are invited to build upon our approach to further enrich the volunteered geographic information in an automated manner.

## Introduction

OpenStreetMap^1^ (OSM) is a community-driven effort to provide free and open access to global spatial data. Volunteered geographic information, which leverages local knowledge to map the geometries and attributes of both natural and urban features, is widely used for humanitarian crises^2,3^, city planning^4^, scientific studies^5,6^, and navigation systems^7^. For example, OSM building footprints as well as streets, roads, rivers, and basic community services have been used to support urban planning and land administration, especially in parts of the world with little traditional data availability^8^.

**Table 1:** Ground truth comparison with OSM data.

Study area	Dataset	Residential	Non-residential	Total
Fairfax	Ground truth	194,491	10,180	20,4671
	OSM (total)	25,129 (12.92%)	2040 (20.03%)	27,160 (13.27%)
	OSM (correct)	24,989 (12.84%)	1961 (19.26%)	26,950 (13.16%)
Mecklenburg	Ground truth	306,700	20,973	327,673
	OSM (total)	28,874 (9.41%)	2625 (12.51%)	31,499 (9.61%)
	OSM (correct)	28,640 (9.33%)	2200 (10.48%)	30,840 (9.41%)
Boulder	Ground truth	20,687	2382	23,069
	OSM (total)	14,140 (68.35%)	1118 (46.93%)	15,258 (66.14%)
	OSM (correct)	14,017 (67.75%)	1006 (42.23%)	15,023 (65.12%)

**Figure 1:**
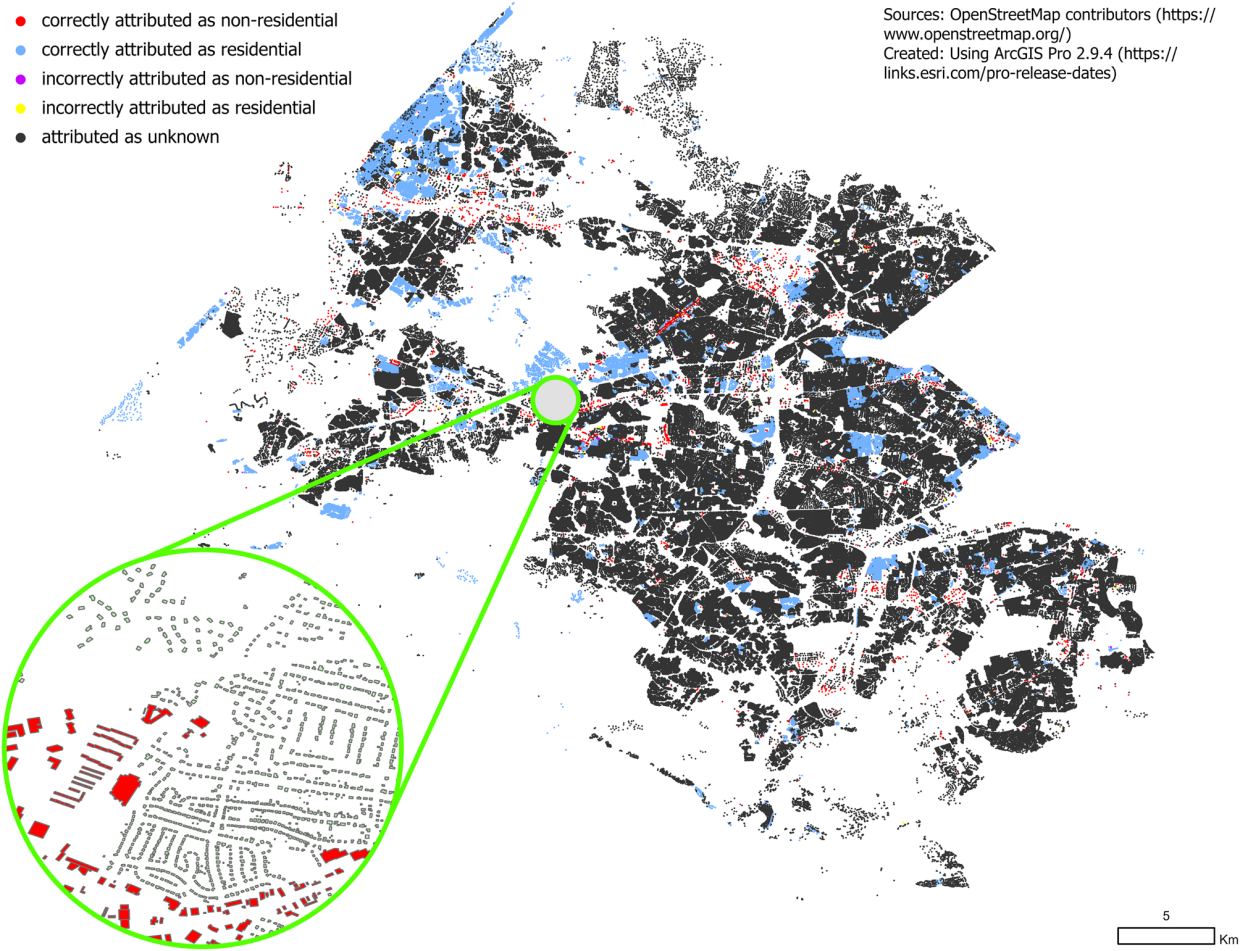
Residential and non-residential building types based on OpenStreetMap data for Fairfax County, USA. Most building types are unknown due to OSM not having explicit information.

For many locations, OSM geometries delineating streets, natural features, and building footprints are highly complete and accurate, often matching or overtaking traditional data sources such as the Central Intelligence Agency (CIA) World Factbook and United States Census Topologically Integrated Geographic Encoding and Referencing (TIGER)/Line data^9–12^. However, even in the data rich locations, the semantic information that records the type and function of these features is very sparse such that the vast majority of features mapped have little to no descriptive attributes.

To illustrate this, Table 1 compares the number of *residential* and *non-residential* buildings in OSM (both the total number and buildings that are correctly classified) with the ground truth data. OSM correctly labels 12.84% of residential and 19.26% of non-residential buildings for Fairfax County, 9.33% of *residential* and 10.48% of *non-residential* buildings for Mecklenburg County, and 67.75% of *residential* and 42.23% of *non-residential* buildings for the City of Boulder. Note that the total number of labeled buildings and the number of correctly labeled buildings for each type are almost the same. Thus, despite the lack of completeness in building type information, the number of misclassified buildings is less than 1%.

Figure 1 further illustrates this by mapping the building footprints in Fairfax County and color coding their accuracy when compared with ground truth data. We observe that in most of the cases where OSM building type information is available, it is correct. However, for the vast majority of buildings, building type information is unknown or unclear. The incomplete nature of the attribute data is a shortcoming that limits the usefulness of OSM data. Therefore, this study proposes a supervised learning approach to add meaningful, semantic information to OSM data without manual intervention. We present a basic demonstration of this approach to classify OSM building footprints by their type as either *residential* or *non-residential*. This particular semantic information is of limited availability at the building footprint level in, both, OSM and official datasets across the United States (US) and globally.

First, we use existing high quality data available through OSM to derive geometric attributes for each building footprint (e.g., area, distance to roads, distance to parking lots, underlying land use) as well as available descriptive attributes in three study areas in the US, as follows: Fairfax County in Virginia, Mecklenburg County in North Carolina, and the City of Boulder in Colorado. Our choice of location has been dictated by available ground truth data and to provide a mix of urban, sub urban and rural areas. Next, based on existing and newly derived building footprint attributes, a set of models are trained to classify the building type using ground truth data obtained from official sources from each study area. Upon comparing to ground truth testing data, we show that our learned models yield high accuracy in all three locations. The learned models from each study area are then transferred to classify building types in alternative study areas. As we will show, this approach again yields high accuracy. The results demonstrate that our approach (1) exhibits high accuracy in regions where authoritative ground truth data is available to train the model, and (2) transfers to new regions for which no ground truth may be available for training. We note that this is just one application of our approach and thus invite the OSM and data science community to build upon it in order to enrich such volunteered geographic information without extensive manual efforts.

**Figure 2:**
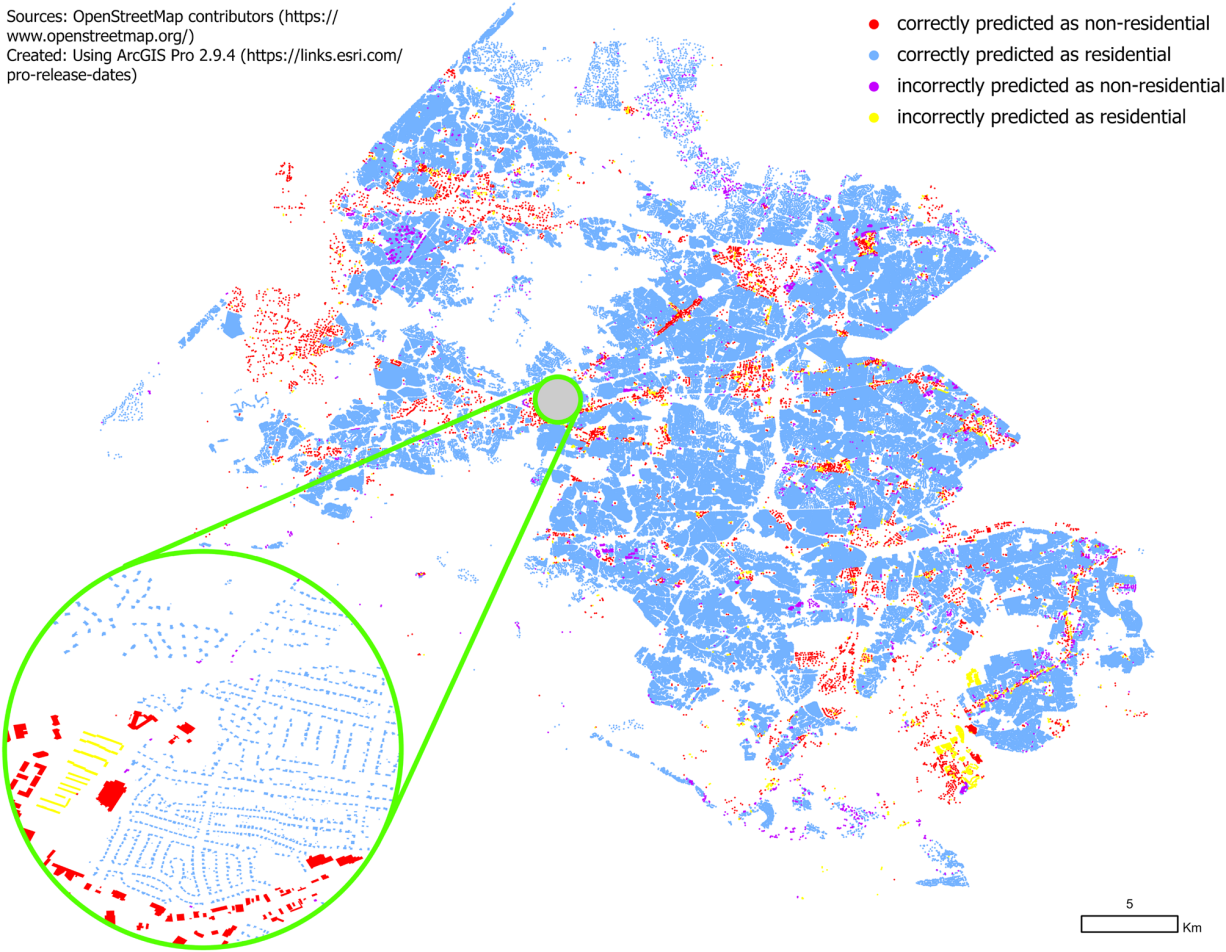
Residential and non-residential building type based on our proposed model for Fairfax County, USA.

## Results

Before the methodology, we first present the main findings of our approach. Figure 2 maps the prediction results for Fairfax County using our proposed supervised classification approach where ground truth data is used for both training and testing (80/20 random split, respectively). We observe that unlike using raw OSM data as shown in Fig. 1, all of the missing data have now been filled with predictions. We can observe visually that most of the building types are correctly classified. Our experiments show an overall accuracy of 98% (see “Experiments”), which is defined as the number of correctly classified buildings divided by the total number of buildings. This implies that only 2% of buildings have their type classified incorrectly. Our experiments also show that the trained model for Fairfax County transfers to other counties with high accuracy (96% when transferred to Mecklenburg County and 93% when transferred to City of Boulder), thus facilitating high-fidelity building type prediction for regions where no ground truth building type data is available. Complete details of the results for each study area are found in the “Experiments” section.

## Methods

Recall that given the sparsity of the semantic information encoded in OSM building data, our objective is to predict the type of buildings based on data available in OSM. Below we describe (1) ground truth and OSM data acquisition for each of the study regions, (2) data processing, (3) feature extraction from OSM building footprints, and (4) building classification. For reproducibility, all our code is available at https://github.com/heykuldip/osm_buildings_classification and a repository of the data used is at https://osf.io/3j46v/.

### Data

We selected three study areas for which we were able to obtain ground truth data including Fairfax County, Mecklenburg County, and the City of Boulder. We included the City of Boulder to examine model differences in a city versus suburban setting (see Table 2). While extremely useful, we note that official data mapping or defining building types is not publicly available for the vast majority of the counties in the US or other regions elsewhere.

**Table 2:** Characterization of the study areas.

Study area	Type	Population	Area (sq mi)
Fairfax County	Suburban	1,150,309	406
Mecklenburg County	Suburban	1,115,482	546
City of Boulder	Urban	108,250	27.366

We used PyOsmium^13^ to extract building polygons from ways and relations elements of OSM. We extracted the building footprints based on whether the ‘building’ tag of OSM polygons have any values. This step is necessary, as OSM include many spatial objects that are not buildings, such as bodies of waters, trees, roads, and intersections.

We downloaded the official building footprint data with associated building types from each administrative unit’s spatial data portal. Ground truth data for Fairfax County was obtained from^14^; ground truth data for Mecklenburg County was obtained from^15^; and ground truth data for City of Boulder was obtained from^16^.

### Data preprocessing

Since our goal is to predict *residential* and *non-residential* building types, we first map a large number of heterogeneous building types in both OSM and the ground truth data (e.g. apartments, church, office) to these two classes. For OSM data, we aggregate building types based on the building tag values to create three meta-categories—*residential, non-residential*, and *unknown* (Table 3). We note that in OSM the *unknown* category is by far the most common, composed mostly of buildings with the tag value ‘yes’. This categorization was used to compare the OSM raw data to the ground truth data to produce Fig. 1.

For the ground truth datasets for Fairfax County, Mecklenburg County, and City of Boulder, which we use to train and validate our models, we aggregate building types based on building tags to create two meta-categories—*residential* and *non-residential* (Table 4). We exclude buildings for which no clear building type is provided or which are not clearly buildings, so as to not compromise our ground truth data (i.e. buildings labeled as building types ‘Mobile Home’, ‘Agricultural’, ‘Foundation/Ruin’, and ‘Misc’). This way, we excluded 2.24%, 0.35%, and 32.33% of total buildings in the Fairfax, Mecklenburg, and Boulder official datasets, respectively.

**Table 3:** OSM building type meta-categories for Fairfax, Mecklenburg, and City of Boulder.

Category	Tag values
Residential	residential, apartments, dormitory, house, semidetached_house
Non-residential	public, commercial, industrial, school, church, office, retail, hotel, warehouse, kindergarten, civic, hospital
Unknown	yes, detached, terrace, garage, roof, shed, parking, garages, greenhouse, static_caravan, service, construction, misc_buildings

**Table 4:** Ground truth building type meta-categories for Fairfax, Mecklenburg, and City of Boulder.

Study area	Residential	Non-residential	Not used
Fairfax	Single Family Residential, Multi-Family Residential	Commercial, Industrial, Public	Mixed Use, Mobile Home, Multi Story Garage, Other
Mecklenburg	Single-Family, Multi-Family, Condo/Townhome	Commercial, Govt-Inst, Hotel/Motel, Office, Warehouse	Warehouse Lg, StadiumArena, Manufactured
Boulder	Residential	Commercial, Industrial, Public, Medical, Public Safety, Religious, School	Agricultural, Foundation/Ruin, Garage/Shed, Parking Structure, Tank, Misc

To find the corresponding buildings in OSM and in the ground truth datasets, we perform a spatial join on the building polygons across the two datasets. Therefore, every building in OSM is mapped to the building in the ground truth data having the largest spatial intersection. Buildings in OSM that do not intersect any building in the ground truth data are removed from our study. For example, Fairfax County has 269,366 official buildings. A join between the official data and the OSM building footprint data results in 197,215 official buildings and 204,672 OSM buildings. The difference can be explained whereby in some cases, many smaller buildings in OSM are contained by one official building. For each of these buildings, we now have both a rich source of data from OSM as well as the ground truth building type obtained from the official sources. In the data pre-processing step, we used the Geopandas^17^ library for geospatial operations on our input data.

### Deriving features for classification

Geometric properties of building footprints and their spatial relationship to other features can be used to predict building type^18–20^. Therefore, we enhance the sparse building attributes found in OSM data by deriving several new geometric attributes based on the shape and location of the building footprints. Below we describe the features, including proximity to roads, proximity to parking lots, building footprint area, intersection with land use, and existing tags, and how these features are obtained from OSM.

### Proximity to roads

The road network is one of the most exhaustive features in OSM that has been used as an effective method for identifying *residential* buildings^21^. We use a similar technique and extend it to predict both the *residential* and * non-residential* class. While many buildings in OSM do not have an explicit building type tag, all road segments in OSM have tags (stored in the ‘highway’ tag of a road segment) indicating the specific road class (e.g., ‘residential’, ‘motorway’, or ‘service’). We hypothesise that this information is a useful predictor to classify the type of nearby buildings.

For this purpose, we enrich each building in OSM with multiple dichotomous indicator variables that discriminate whether or not each building falls in range of four road meta-categories: (1) residential roads, (2) highways, (3) motorways, and (4) service roads. The OSM ‘highway’ tag defines the road types according to their types and capacities, varying from pathways to expressways. The road type tags in OSM map to our meta-categories as follows: (1) *Residential Roads*: Using tag values ‘residential’ and ‘living_street’; (2) *Highways*: Using tag values ‘primary’, ‘secondary’ and ‘tertiary’; (3)* Motorways*: Using tag values ‘motorway’ and ‘trunk’; (4) *Service roads*: Using tag value ‘service’.

For each meta-category of roads, we add three indicator attributes to each building, where a value of 1 indicates that the building is located in a 0–30 m, 0–60 m, and 0–90 m range of the road network and a value of 0 indicates that it is not. This yields a total of twelve indicator attributes for each building where indicators 0 to 3 correspond to residential roads, 4 to 6 to highways, 7 to 9 to motorways, and 10 to 12 to service roads. For example, the indicator values [1, 1, 1, 0, 0, 0, 0, 0, 0, 0, 0, 1] indicate that a building falls within a 0–30 m radius of a residential road (indicated by the first indicator variable) and thus, also in a 0–60 m and 0–90 m radius (indicated by the second and third indicator variable). The building is not in range of any highways or motorways. However, the building falls within 0–90 m distance of a service road (indicated by variables twelve), but not within 0–30 m or 0–60 m.

To efficiently compute the indicator variables for each building, we create corresponding buffers (of 0–30 m, 0–60 m, and 0–90 m) around each road in OSM. Then, we perform a spatial join between these buffers and the polygons of the buildings in OSM. For each intersection, depending on the ‘highway’ tag of the road, the corresponding indicator variable (using the mapping above) is set to 1.

### Proximity to parking lots

We hypothesize that distance from parking lots of various sizes can be used to predict building type. For example, we would expect that as parking lot size increases, the likelihood that the building is a *non-residential* building would also increase. We extract the parking lot geometries from the OSM data using the ‘amenity’ tag having a value of either ‘parking’ or ‘parking_space’. We first examine the distribution of parking lot size across the study region and create three classes of parking lots based on the natural breaks of the parking lot size distribution using the Fisher-Jenks algorithm^22^.

Next, we enrich each building in OSM with parking lot indicator variables that indicate whether or not each building falls in a 30 m, 60 m, or 90 m range of three parking lot categories: (1) small, (2) medium, and (3) large, yielding a total of nine additional indicator variables. To compute the parking lot indicator variables for each building, we create corresponding buffers around the parking lots. We then perform a spatial join between these buffers and the polygons of the buildings in OSM.

### Building footprint size

The size of a building footprint can a key predictor of building type^23^. Therefore, in addition to the road network and parking lot buffers, we compute the area based on the building footprint geometry and use the area as another (ratio-scaled) feature for our decision tree model.

### Intersection with land use

OSM data includes the geometries and descriptive attributes for different underlying land use upon which the buildings are located. This data may explicitly contain information on the use of the land that the buildings are built on, thus providing insight into the use of the building itself^24^. Therefore, we extracted polygons having the ‘landuse’ tag in the OSM data and spatially joined them with the building footprints, resulting in another feature for our machine learning model.

### OSM building tags

In addition to geometry, each building has a set of associated tags, which describe features using pairs of unique keys and corresponding values. Besides the above derived features, we utilized the tags from the OSM data that we deemed relevant for accurately categorizing the buildings. The tags are: ‘building’, ‘name’, ‘source’, ‘addr:street’, ‘building:levels’, ‘shop’, ‘website’, ‘brand’, and ‘amenity’. With the exception of the ‘building’ tag, each of the tags themselves are treated as a binary indicator variable where buildings have a value of 0 if they do not have a tag and 1 if they do. For the ‘building’ tag, we utilize the tag value rather than the presence or absence of the tag itself and encode each of the values as a nominal indicator variable. Since there are theoretically an infinite number of building tag values, we select the most common values, namely the values ‘apartments’, ‘church’, ‘civic’, ‘commercial’, ‘construction’, ‘detached’, ‘dormitory’, ‘garage’, ‘garages’, ‘greenhouse’, ‘hospital’, ‘hotel’, ‘house’, ‘industrial’, ‘kindergarten’, ‘office’, ‘parking’, ‘public’, ‘residential’, ‘retail’, ‘roof’, ‘school’, ‘semidetached_house’, ‘service’, ‘shed’, ‘static_caravan’, ‘terrace’, ‘warehouse’, and ‘yes’. We create a separate nominal variable called ‘miscellaneous’ that includes all the remaining unique building values across the three study areas.

In general, we manually selected these tags based on their relevance to distinguish building types while making sure that the model is capable of transfer learning independently of any geographic area. For example, if a building contains a website address, it seems more likely to be classified as ‘non-residential’. It is worth noting, however, that our model is flexible to handle any tags available in the OSM raw data, the hand-picked tags are a proof-of-the-concept of our proposal.

### Decision tree classification

Using the features described in the previous sections, we use a classic C4.5 binary decision tree classifier^25^ to recursively find the attributes that yield the highest information gain to construct the decision tree. To train the decision tree, we use the authoritative ground truth building type obtained from the respective counties and city. Our choice of using a decision tree for classification was made due to it’s interpretability, allowing us to understand where and why classification errors are made to guide our search for discriminatory features to separate the *residential* and *non-residential* classes. To parameterize our decision tree, we use Gini-index^26^ which is commonly used as a measure of impurity between classes. We prune the decision tree when no additional decision criterion increases the impurity of a node by no more than 0.01%.

## Experiments

**Figure 3:**
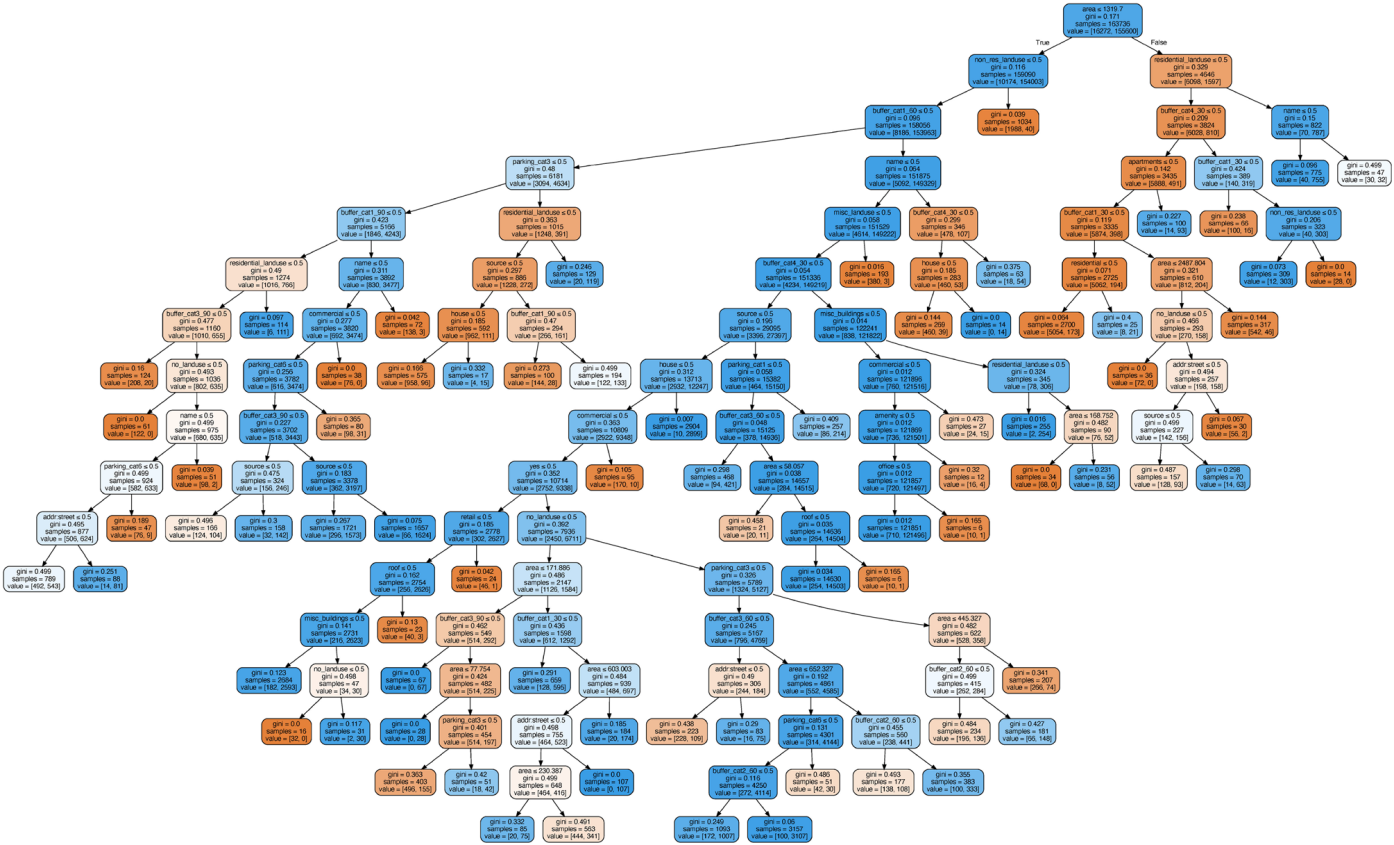
The full decision tree for Fairfax County. Each node specifies the purity of a node measured using the Gini coefficient, the number of buildings (samples) in the node, and the corresponding distribution of types (non-residential, residential). Internal (decision) nodes also specify the attribute which is used to split the node. The color of a node corresponds to class distribution of a node, having mostly non-residential nodes in red, and mostly residential nodes in blue. A high resolution electronic version of this image (to allow zooming and scrolling) can be found in our Github repository at https://github.com/heykuldip/osm_buildings_classification.

### Qualitative analysis of the decision tree model

Figure 3 shows the resulting decision tree for Fairfax County, using a 80% random sample of all buildings having a total of 171,872 building out of which 16,272 are *non-residential* and 155,600 are *residential*. This decision tree has a total of 148 nodes including 72 leaf nodes. Note that the root of the tree starts by using the area of the building, indicating that it is the most discriminating feature. This result confirms existing work, which has shown that the size of the building footprint area is an important predictor of a building type^23^. Specifically, the decision-tree first checks whether the area of the building is less than 1319.7 square meters. Given that there is a larger proportion of residential buildings in the group with the small building footprints (10,174 *non-residential* and 154,003 *residential* buildings), the decision-tree learns that smaller buildings tend to be *residential*. In contrast, given that there is a larger proportion of *non-residential* buildings in the group with the larger building footprints (6098 *non-residential* and 1597 *residential* buildings), the decision-tree learns that larger buildings tend to be *non-residential*.

In both resulting branches, the decision tree then checks the ‘landuse’ tag, confirming our intuition that the type of land use is a useful predictor of the type of a building. But it is nowhere near sufficient to classify the type of all buildings. The decision tree learns that buildings that are (1) smaller than 1319.7m$^2$ and (2) are of residential land use tend towards *residential* buildings (8186 *non-residential* and 153,963 *residential* buildings). However, in the case that buildings are (1) smaller than 1319.7m$^2$ and (2) are of *non-residential* land use tend to be *non-residential* buildings (1988 *non-residential* and 40 *residential* buildings). In fact, for this branch, the decision tree concludes that other attributes no longer provide sufficient reduction of impurity (as measured by Gini index) of this node, thus that this node is a leaf node, thus predicting all such buildings as *non-residential*.

Other branches are longer, up to a length of 17. An important branch of this tree is the branch in which the decision tree learns that buildings that are (1) small, (2) of non-residential land use, (3) are not within a 60 m range of Category 1 (residential) roads, (4) do not have a building name, (5) do not have ‘misc’ land use, (6) are not within a 60 range of a Category 4 (service) road, (7) are not have the value ‘miscellaneous’ in the ‘building’ tag, (8) does not have the value ‘commercial’ in the building tag, (9) does not have the value ‘amenity’ in the building tag, and (10) does not have the value ‘office’ in the building tag tend towards *residential* buildings (710 *non-residential* and 121,496 *residential* buildings). Based on the Gini index, the decision tree decides this node to be a leaf node, thus classifying such buildings as *non-residential* buildings. The interested reader may refer to the high resolution complete decision tree in our Github repository at https://github.com/heykuldip/osm_buildings_classification for Fairfax County to understand which attributes and their values guide this decision tree model.

### Evaluation metrics

To quantitatively evaluate our model, we first measure the *accuracy* of the classification, defined as the fraction of correctly classified building types across all buildings. However, some of the study regions such as Fairfax County have a class imbalance, where the number of *residential* buildings far outweigh the number of *non-residential* buildings (194,491 *residential* versus 10,180 *non-residential*, see Table 1). Due to this class imbalance, using only accuracy as a measure can be misleading, as a naive approach that classifies all buildings as *residential* would already have an accuracy of $\frac{194,491}{194,491+10,180}=0.9503$. We additionally want to understand how well our model is able to predict *non-residential* buildings. For this purpose, we compute three measures that capture the ability of the models to predict the two classes (*residential* and *non-residential*):

**Table 5:** Prediction results.

Study area	Class	Precision	Recall	$F_1$-score	Accuracy﻿	Avg﻿ $F_1$-score
Fairfax	Non-residential	0.8122	0.7851	0.7984	0.9802	0.8940
	Residential	0.9887	0.9905	0.9896		
Mecklenburg	Non-residential	0.8081	0.6981	0.7491	0.9696	0.8664
	Residential	0.9792	0.9885	0.9838		
Boulder	Non-residential	0.8571	0.8233	0.8399	0.9673	0.9108
	Residential	0.9795	0.9840	0.9818

1. The *precision* of a class, defined as the number of buildings correctly predicted as that class divided by the total number of buildings predicted as that class. Intuitively, the precision of class corresponds to the probability that a building that is predicted to have class *X* actually has class *X*.
2. The *recall *of a class, defined as the number of buildings correctly predicted as that class divide by the total number of buildings in that class (in the ground truth). Intuitively, the recall of a class corresponds to the probability that a building have class *X* is correctly classified as class *X*.
3. The $F_1$*-score* of a class, which is the harmonic mean of precision and recall of a class.

### Building type prediction results

Recall that in the “Results” section, we presented the high level results for one of the models that was trained and tested on Fairfax County as an example. Here we describe the results for Fairfax County and the other two study regions in detail. Table 5 shows prediction results for each of the three models that were trained and tested on the authoritative ground truth data. To evaluate each model, we use 80% of buildings (chosen uniformly at random) as the training set for which to build the decision tree, and the remaining 20% of buildings as the test set. Table 5 shows our evaluation metrics and Fig.  2 shows the results for Fairfax County. All remaining maps presenting the prediction results can be found in the Supplemental Materials.

We observe that Fairfax County has the highest accuracy at $98.02\%$ followed by Mecklenburg County ($96.96\%$) and City of Boulder ($96.73\%$). However, by looking at precision and recall for individual classes, we observe that the high accuracy for Fairfax County is attributed to the high precision and recall ( $99.0\%$) for the *residential* class. This comes at a cost for the *non-residential* class, having a precision of $81.22\%$ and implying that almost $20\%$ of buildings predicted as *non-residential* are predicted incorrectly. We also observe a recall of only $79.84\%$, meaning that more than one in five *non-residential* buildings are incorrectly predicted as *residential* buildings. For City of Boulder, where the overall accuracy is lower than for Fairfax County, we see that the *non-residential* class is classified more accurately, indicated by higher precision and recall values. Summarizing, we observe that our model is very accurate at predicting residential buildings, evident by an $F_1$-score of nearly $99\%$ for the *residential* class. However, our model does make more errors predicting the *non-residential* class, evident by $F_1$-score between 74.91 and $83.99\%$ across the three study regions for the *non-residential* class.

**Figure 4:**
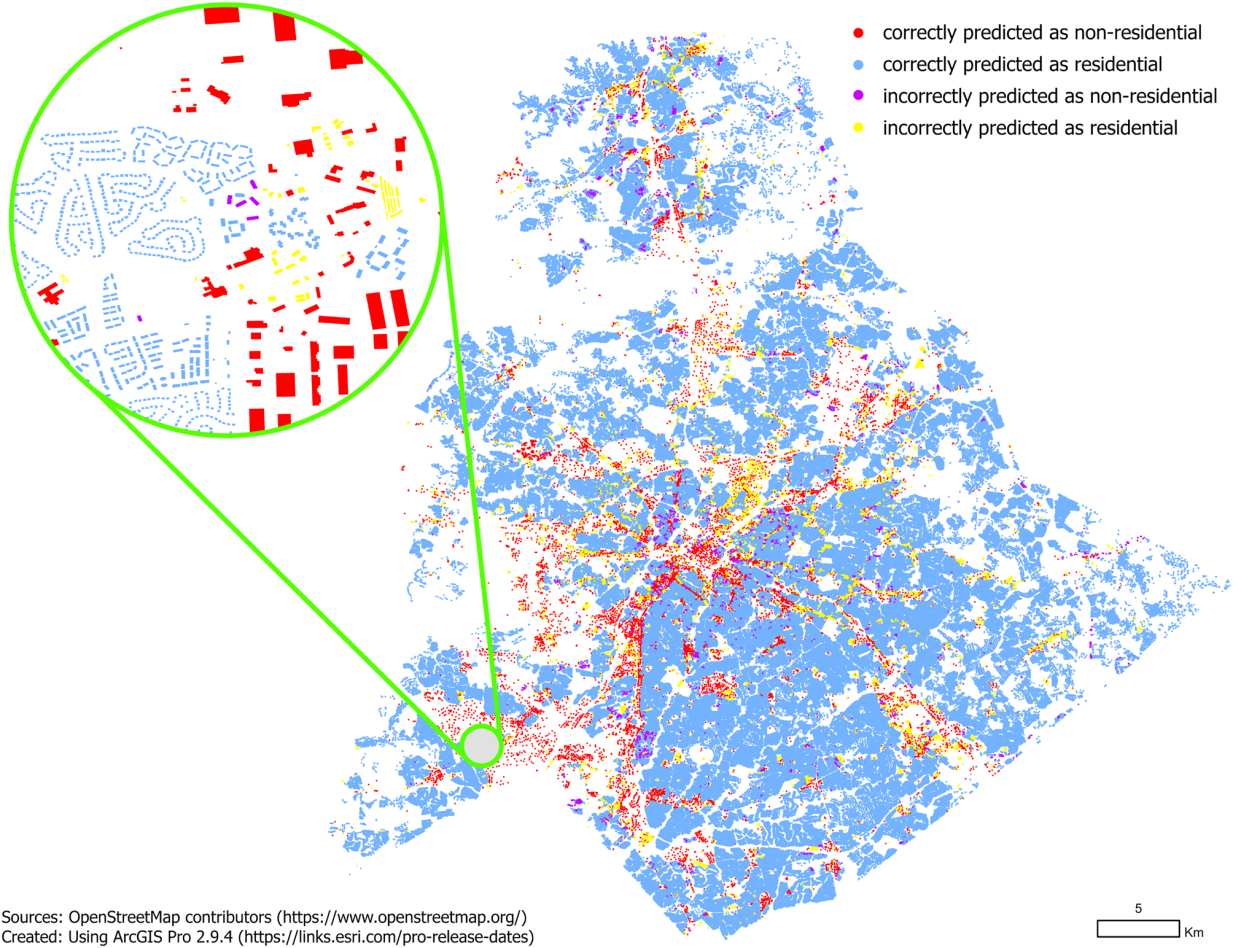
Residential and non-residential building type based on transfer learning model for Fairfax County transferred to Mecklenburg County.

### Transfer learning results

Our study was motivated by the fact that it is hard to find descriptive attributes (such as building type) in both, official and OSM data sources^27,28^. This type of data is commonly used for a variety of applications ranging from geospatial modeling and analysis and urban planning applications. To this end, we designed our pre-trained classification model so that it can be applied to any study area, requiring only OSM data as an input. To demonstrate the effectiveness of the pre-trained model, we tested each of the three models (trained on all data available for the respective study region) on the alternative study areas. The result of the transfer learning experiments (that transfer the model trained on one region to another region) are presented in Table 6. The transfer learning results for the model trained on Fairfax and tested on Mecklenburg County are presented in Fig. 4. All remaining maps presenting transfer learning results can be found in the Supplemental Materials.

Overall, we observe that transferring models reduces their accuracy and $F_1$-scores. While the Fairfax model had an accuracy of 0.9802 (Table 6), the accuracy drops to 0.9645 and 0.9253 when applied to Mecklenburg County and City of Boulder, respectively. Despite the drop in accuracy, it is a very promising result, showing that for Mecklenburg County, more than $96\%$ of buildings are classified correctly. Thus, even if no authoritative ground truth data was available in Mecklenburg County (as is the case for most regions in the US and across the world), our model learned in Fairfax County and applied to Mecklenburg County still yields very good building type classification results.

Looking at precision and recall for individual classes, we observe that the predictions for the *residential* class are still very good, with $F_1$-scores of 0.9812 and 0.9581 for Mecklenburg County and City of Boulder, respectively. It is a particularly important result for urban planning applications that require an accurate estimation of the residential buildings. However, precision and recall further drop for the *non-residential* class, having values between 0.5929 and 0.7704 for the models trained in Fairfax County and Mecklenburg County. For the model trained for the City of Boulder, the results of transferring the model to Fairfax and Mecklenburg County are substantially worse. We mainly attribute this bad performance to the small size of the City of Boulder dataset, having an order of magnitude fewer buildings (see Table 1). Due to the much lower number of buildings, the model is not able to generalize as well as the Fairfax and Mecklenburg County models. Summarizing, we see that the rules learned by the decision tree models trained in Fairfax County and Mecklenburg county generalize well and can be applied to high accuracy to other counties. It implies that our model can be used to obtain high accuracy building type maps for any county or city—at least in the United States.

**Table 6:** Transfer learning results.

Training dataset	Test dataset	Class	Precision	Recall	$F_1$-score	Accuracy	Avg $F_1$-score
Fairfax	Mecklenburg	Non-residential	0.8005	0.5929	0.6812	0.9645	0.8312
		Residential	0.9726	0.9899	0.9812		
	Boulder	Non-residential	0.6235	0.6982	0.6587	0.9253	0.8084
		Residential	0.9648	0.9515	0.9581		
Mecklenburg	Fairfax	Non-residential	0.6236	0.7375	0.6758	0.9648	0.8286
		Residential	0.9861	0.9767	0.9814		
	Boulder	Non-residential	0.6046	0.7704	0.6775	0.9243	0.8173
		Residential	0.9727	0.9420	0.9571		
Boulder	Fairfax	Non-residential	0.4125	0.7838	0.5406	0.9337	0.7524
		Residential	0.9881	0.9416	0.9643		
	Mecklenburg	Non-residential	0.5026	0.7651	0.6067	0.9365	0.7861
		Residential	0.9833	0.9482	0.9655

**Table 7:** Ablation study results.

Experiment	Study area	Class	Precision	Recall	$F_1$-score	Accuracy	Avg $F_1$-score
‘building’ tag only	Fairfax	Non-residential	0.9595	0.2062	0.3394	0.9595	0.6593
		Residential	0.9595	0.9995	0.9791		
	Mecklenburg	Non-residential	0.8218	0.1471	0.2496	0.9425	0.6098
		Residential	0.9439	0.9978	0.9701		
	Boulder	Non-residential	0.9375	0.4582	0.6156	0.9391	0.7913
		Residential	0.9392	0.9964	0.9669		
‘building’ tag and selected tags	Fairfax	Non-residential	0.8596	0.3774	0.5245	0.9661	0.7535
		Residential	0.9685	0.9968	0.9824		
	Mecklenburg	Non-residential	0.8545	0.2475	0.3838	0.9502	0.6789
		Residential	0.9520	0.9972	0.9741		
	Boulder	Non-residential	0.8781	0.5806	0.6990	0.9476	0.8351
		Residential	0.9527	0.9906	0.9713		
‘building’ tag, selected tags, and landuse polygons	Fairfax	Non-residential	0.9145	0.5150	0.6590	0.9740	0.8227
		Residential	0.9757	0.9975	0.9865		
	Mecklenburg	Non-residential	0.9160	0.4397	0.5942	0.9620	0.7871
		Residential	0.9635	0.9973	0.9801		
	Boulder	Non-residential	0.8712	0.6543	0.7474	0.9534	0.8608
		Residential	0.9605	0.9886	0.9743		
‘building’ tag, selected tags, landuse polygons, and footprint area	Fairfax	Non-residential	0.8040	0.6989	0.7478	0.9765	0.8677
		Residential	0.9843	0.9911	0.9877		
	Mecklenburg	Non-residential	0.8237	0.6130	0.7029	0.9664	0.8425
		Residential	0.9736	0.9909	0.9822		
	Boulder	Non-residential	0.8093	0.7925	0.8008	0.9588	0.8889
		Residential	0.9759	0.9782	0.9770		
‘building’ tag, selected tags, landuse polygons, footprint area, and distance to roads and parkings lots	Fairfax	Non-residential	0.8122	0.7851	0.7984	0.9802	0.8940
		Residential	0.9887	0.9905	0.9896		
	Mecklenburg	Non-residential	0.8081	0.6981	0.7491	0.9696	0.8664
		Residential	0.9792	0.9885	0.9838		
	Boulder	Non-residential	0.8571	0.8233	0.8399	0.9673	0.9108
		Residential	0.9795	0.9840	0.9818		

### Ablation study results

Our proposed solution combines multiple building features extracted from OSM, including user-specified tags, land use polygons, area of building footprints, and distance distance from roads and parking lots. In the following experiment, we aim at understanding which of these features has the highest predictive power to classify building types. For this purpose, we evaluate the results by iteratively turning individual features on. Table 7 shows the results of this experiment. We first run a baseline experiment which only uses the ‘building’ tag information and builds a decision tree only based on the values of this tag. For this straightforward model, we observe an overall accuracy of 0.9595, which is high. However, we observe that this high accuracy is mainly a result of simply classifying all buildings as *residential*. This is evident by the low recall of as low as 0.1471 in Mecklenburg county of the *non-residential* class, indicating that more than $85\%$ of *non-residential* buildings in Mecklenburg County are incorrectly classified as *residential* based on ‘building’ tags only. A measure that treats the two classes equally despite their imbalance, is the average $F_1$ score, only 0.6098 for Mecklenburg County in this experiment, which is only slightly higher than a uniformly random choice (flipping a coin for each building) that would have an average $F_1$-score of 0.5.

In the next experiment, we increase the complexity of the decision tree model by adding other selected tags (such as ‘name’, and ‘website’—see our “Methods” section under paragraph OSM Building Tags) to train the decision tree. We observe that this feature significantly increases all metrics, in particular for the *non-residential* class, which confirms out intuition that the presence of additional tags helps to identify *non-residential* buildings. Adding landuse information further increases all metrics, confirming our hypothesis that in cases where no building information is available, the landuse information of the land the building is built is useful as a proxy. We then observe that adding the footprint area (in square meters) of a building does not substantially increase the overall accuracy, but it does increase the average $F_1$-score by allowing to identify more *non-residential* buildings. Looking at our decision tree in Fig. 3, we observe that the model learns that very large buildings (larger than 1319.7 m$^2$) are mostly *non-residential*, which appears to be an intuitive rule learned by the decision tree. Finally, we also include information on the distance to roads and parking lots (as described in the “Methods” section) to obtain our full model, which yields a very high boost in all metrics.

### Comparison with other classification methods

We chose decision trees as our classification method due to their interpretability. Yet, an open question is whether other classification algorithms may perform better and thus, have a higher accuracy and $F_1$ score in classifying building types. To answer this question, we compared the decision tree classifier with k-nearest neighbors classification^29^ having $k=10$, naive bayes classification^30^ assuming Gaussian distributed conditional distributions, random forest^31^, support vector machines^32^, and a single layer perceptron^33^ as a representative of a neural network model. Results for the comparisons are presented in Table 8. The results show that for our use-case of building type classification decision trees, in addition to being easily interpretable, yield results comparable to other classification paradigms.

**Table 8:** Comparison of decision tree with other models using Fairfax data.

Model	Class	Precision	Recall	$F_1$-score	Accuracy	Avg $F_1$-score
Decision tree	Non-residential	0.8122	0.7851	0.7984	0.9802	0.8940
	Residential	0.9887	0.9905	0.9896		
K-nearest neighbors	Non-residential	0.7824	0.4092	0.5374	0.9645	0.7594
	Residential	0.9694	0.9940	0.9815		
Gaussian naive Bayes	Non-residential	0.7506	0.6421	0.6921	0.9720	0.8387
	Residential	0.9817	0.9890	0.9853		
Random forest	Non-residential	0.8410	0.6994	0.7637	0.9791	0.8764
	Residential	0.9849	0.9933	0.9891		
Support vector machine (SVM)	Non-residential	0.9293	0.6433	0.7603	0.9799	0.8749
	Residential	0.9817	0.9975	0.9895		
Linear perceptron classifier	Non-residential	0.7388	0.5967	0.6602	0.9701	0.8225
	Residential	0.9795	0.9892	0.9843		

## Related work

Several studies have been conducted to address the OSM buildings classification problem. While some of the studies are specifically done for building footprints enrichment by complementing OSM with additional data sources^19,20,23,27^, others use the classified buildings for tasks such as population estimation^34^, medical interventions^35^, and semantic maps^36^. An urban morphology analysis-based approach is proposed for building types estimation that derives the correlations among geometries of the building footprints and their types, and a set of rules are established to classify OSM buildings into six categories^18^. Furthermore, the morphological analysis presented in this work also validated the hypotheses that buildings share attributes, such as type, if their footprints are similar geometrically and are closely located. Our model combines these geospatial characteristics with other contextual features, such as proximity to roads and parking lots, to learn the building types in an automated way, addressing the scalability and rigidness challenges of the rule-based technique while achieving accuracy of 0.9802% compared to 0.8577 % of this model. The building classification problem is also addressed in the public health domain using the attributes of buildings’ structures^37^. However, the lack of comparative results with authoritative ground truth data limits the practical usability of this model.

Machine learning approaches are successfully employed to extract building footprints from satellite and aerial images^38,39^ and classify buildings from remote sensing imagery^40–43^, Google Earth images^44^, and light detection and ranging (LiDAR) data^45,46^. Semantic analysis is also coupled with the random forest method to classify urban buildings from images into finer categories^47,48^. A natural language processing (NLP)-based approach is used to classify point-of-interest (POI), land use, and roads data extracted from Baidu Maps that can infer building types^49^. Similar methods are used for correcting OSM building annotations^50^, street labels predictions^51^, autonomous robot navigation^52^, 3D building models^53^, and land cover classification^54,55^. However, these approaches either use region-specific or proprietary datasets that are hard to obtain for applying the models in different places. Our model relies only on the OSM features, eliminating the bottleneck of unavailability or incompatibility of additional data for certain regions. Therefore, the novelty of our approach lies in incorporating geometric, topological, and non-spatial features including distance to surrounding roads of different types, distance to parking lots of different sizes, underlying land use, the area of a building polygon, and a wide variety of user-generated OSM tags.

## Discussion

Previous work shows that attributes such as shape and area are effective for building classification^18^. Our hypothesis is that by combining multiple attributes derived from OSM data, we can improve the quality of such a classification. Thus, we extracted various spatial and non-spatial features of buildings with surroundings and trained a decision tree classier to interpret the generated rules. We corroborated the effectiveness of our approach by applying our model to Fairfax County, Mecklenburg County, and City of Boulder, and comparing the results with corresponding ground truth data. An important outcome of this study is that our model transfers, thus allowing to apply a model learned in one region to a different region, while maintaining high accuracy. Specifically, we observe that a model learned using data from Fairfax County achieves an accuracy of 96.4% when applied to buildings in Mecklenburg County. This result indicates that the models learned in the three regions for which building type data is publicly available can be used for other regions where no authoritative ground truth data is available.

Our analysis shows that although OSM building footprints coverage is extensive, the geometries are still incomplete, observed in cases where building footprints exist in authoritative data sources but not in OSM data. For example, the ground truth data we used from Fairfax County Geographic Information System (GIS) and Mapping Services has 269,366 buildings, but OSM data for the same region includes only 204,672 buildings, which means  24% of buildings are still missing in OSM. Other studies also highlight the incompleteness of OSM buildings compared to authoritative data sources^56–58^. Furthermore, it remains unclear how well our proposed building type classification model transfers to regions and cities outside of the United States. As OSM data is available globally, and tags of buildings, road types, and parking lots are available globally, we theorize that our models should be applicable globally. But further studies using regions outside the United States is needed.

## Conclusion

The lack of standards for OSM user attribute tags and values results in sparse and heterogeneous attribute information, limiting the usage of otherwise rich and accurate OSM data for a variety of applications. Using the approach developed in this work, we can enrich existing OSM data and achieve complete and highly accurate semantic information for such data. Specifically, we designed a supervised learning model that uses OSM raw data to predict building types as *residential* or *non-residential*. Our solution for classifying building types broadly using OSM data has applications in systems that use OSM data, for example for semantic mapping^59^. By enriching OSM data with accurate building types, our model improves the input data for such applications.

For this work, we chose decision trees to allow for easy interpretability of the resulting classification models. Future work may investigate whether more sophisticated classification methods, including deep learning models^60^, may further improve building type prediction. Additionally, we formulated the classification of buildings as a two-class problem and predicted the labels according to their functionality. It could limit the usefulness of our approach, especially for the *non-residential* class, which can be further classified as *commercial*, *industrial*, *public*, etc. As a future line of research, we are interested in extending this work to a multi-class classification problem. We hope that the OSM and data science communities will support this automated enrichment effort and increase the value and usefulness of volunteered geographic information. There is a lot of ‘intrinsic’ information in OSM that is waiting to be uncovered!

## Supplementary Information

Supplementary Information.

## Data Availability

Data are available from OSF at https://osf.io/3j46v/.

## Code availability

The code is available in a GitHub repository at https://github.com/heykuldip/osm_buildings_classification.
