# Supplementary material for: Predicting building types using OpenStreetMap
Source: Sci Rep. 2022 Nov 20;12:19976. doi: 10.1038/s41598-022-24263-w (PMC9676186; doi:10.1038/s41598-022-24263-w)
Supplement: Supplementary file 1 — Supplementary Information. [file 41598_2022_24263_MOESM1_ESM.pdf]

Predicting building types using OpenStreetMap

Kuldip Singh Atwal, Taylor Anderson, Dieter Pfoser, Andreas Züfle

1 Supplementary information

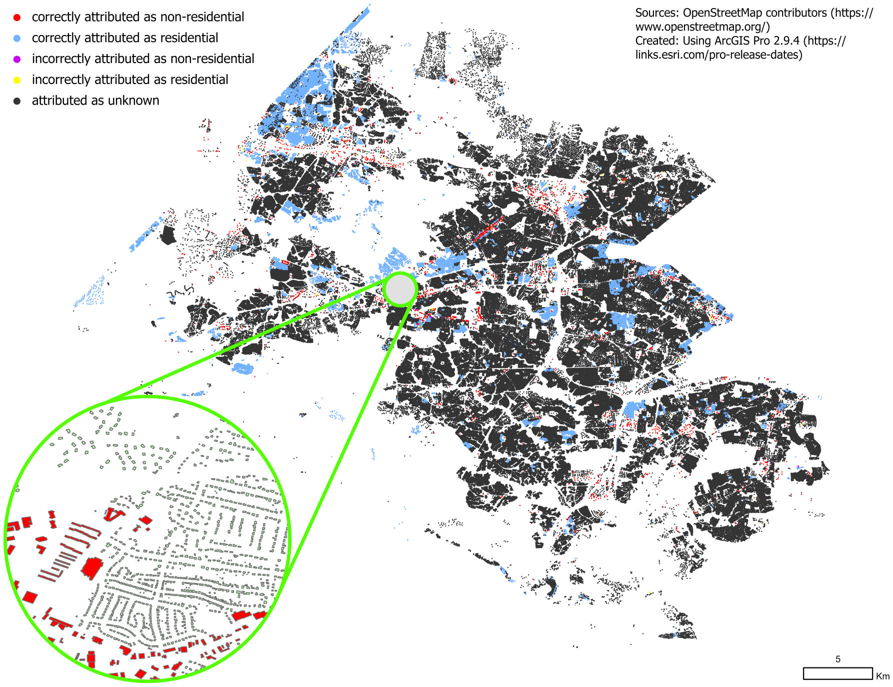

Figure 1: Residential and non-residential building type based on raw OpenStreetMap data for Fairfax County, USA. Most building type are unknown due to OSM not having explicit information.

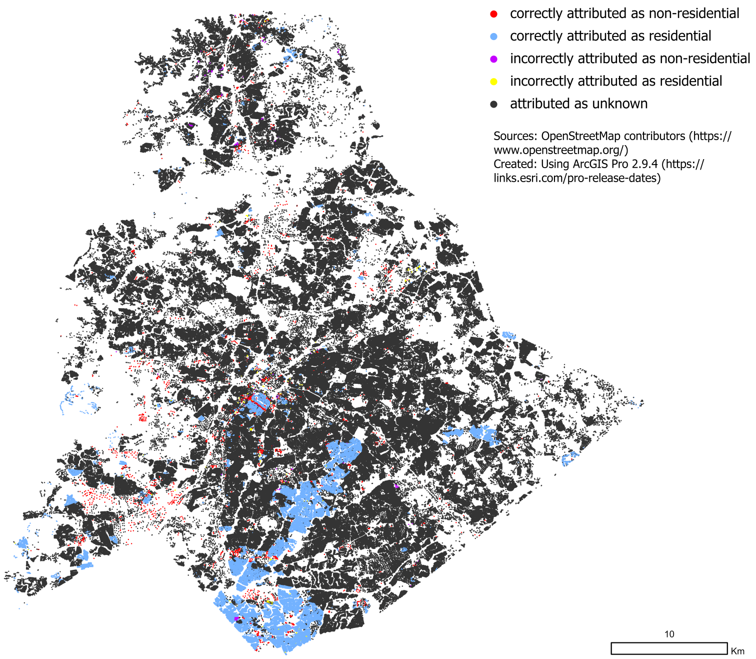

Figure 2: Residential and non-residential building type based on raw OpenStreetMap data for Mecklenburg County, USA. Most building type are unknown due to OSM not having explicit information.

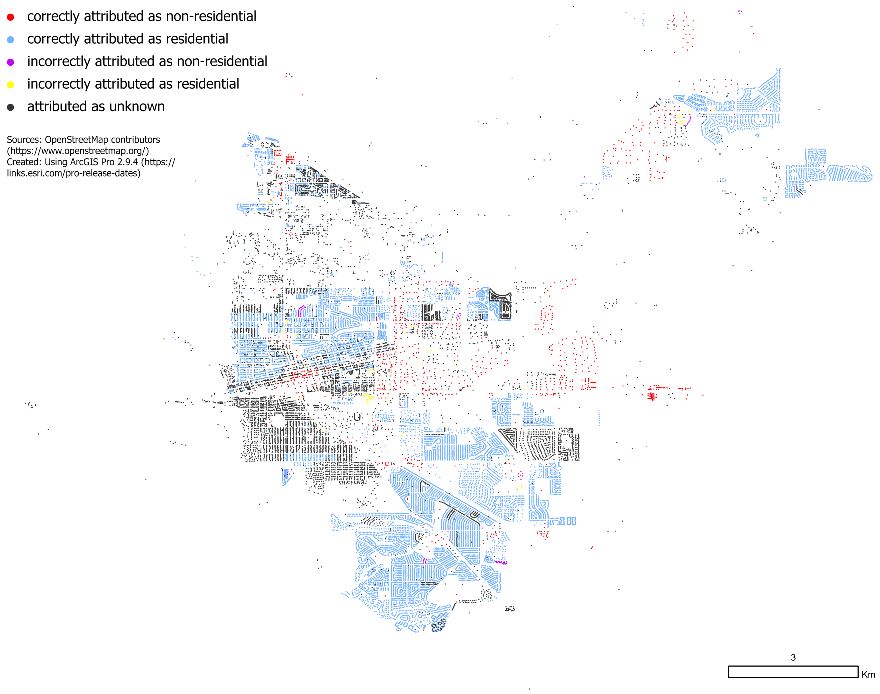

Figure 3: Residential and non-residential building type based on raw OpenStreetMap for City of Boulder, USA. Many building type are unknown due to OSM not having explicit information.

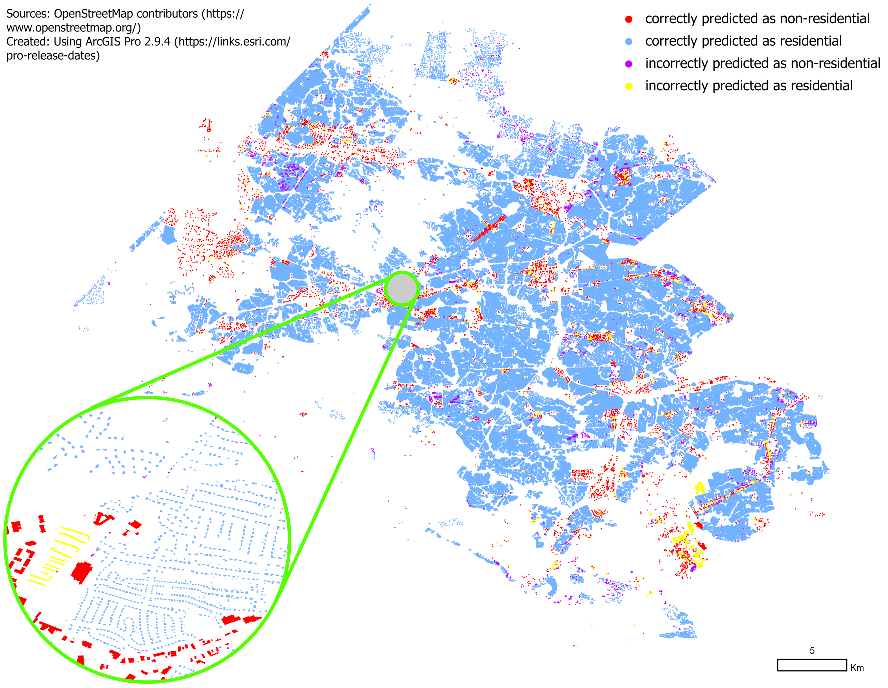

Figure 4: Predictions for residential and non-residential building type for Fairfax County, USA obtained from a decision tree model that was trained and tested on Fairfax County ground truth data.

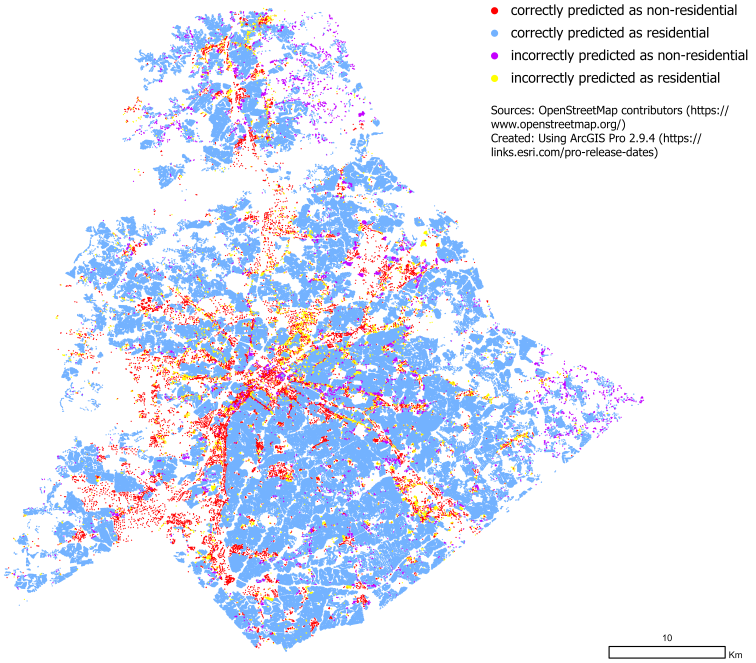

Figure 5: Predictions for residential and non-residential building type for Mecklenburg County, USA obtained from a decision tree model that was trained and tested on Mecklenburg County ground truth data.

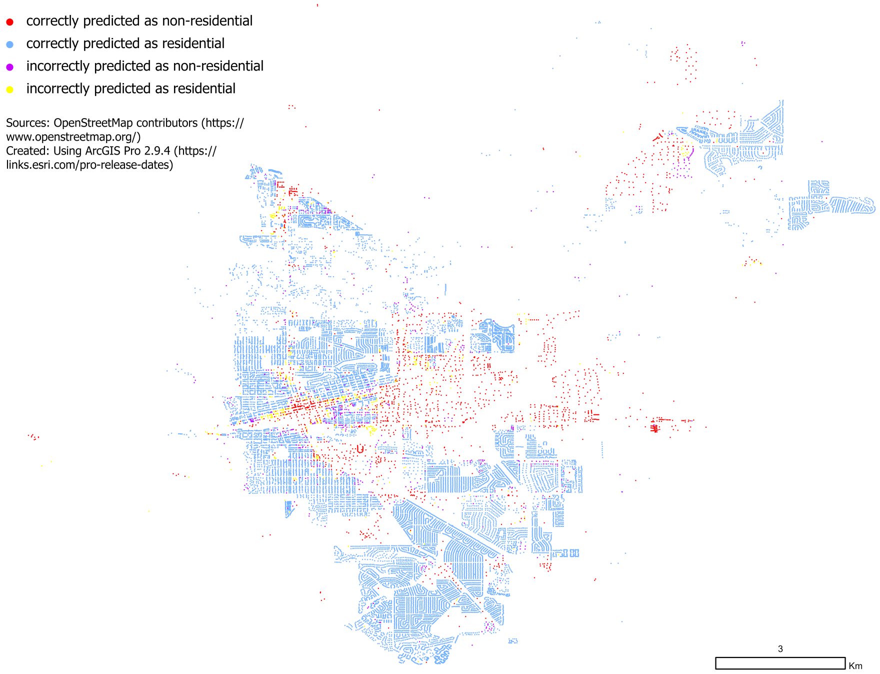

Figure 6: Predictions for residential and non-residential building type for City of Boulder, USA obtained from a decision tree model that was trained and tested on City of Boulder ground truth data.

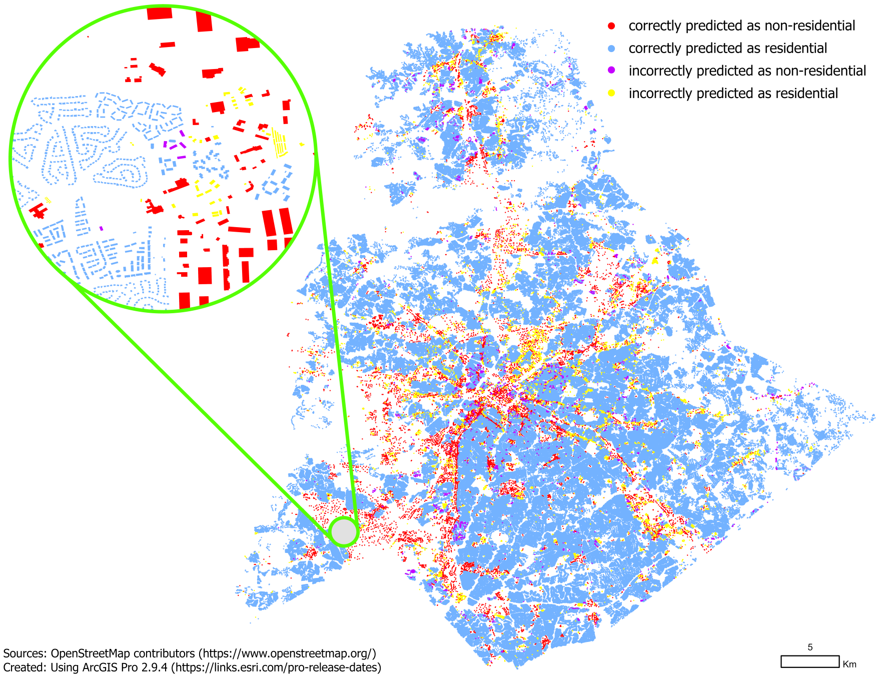

Figure 7: Residential and non-residential building type based on transfer learning model for Fairfax County transferred to Mecklenburg County.

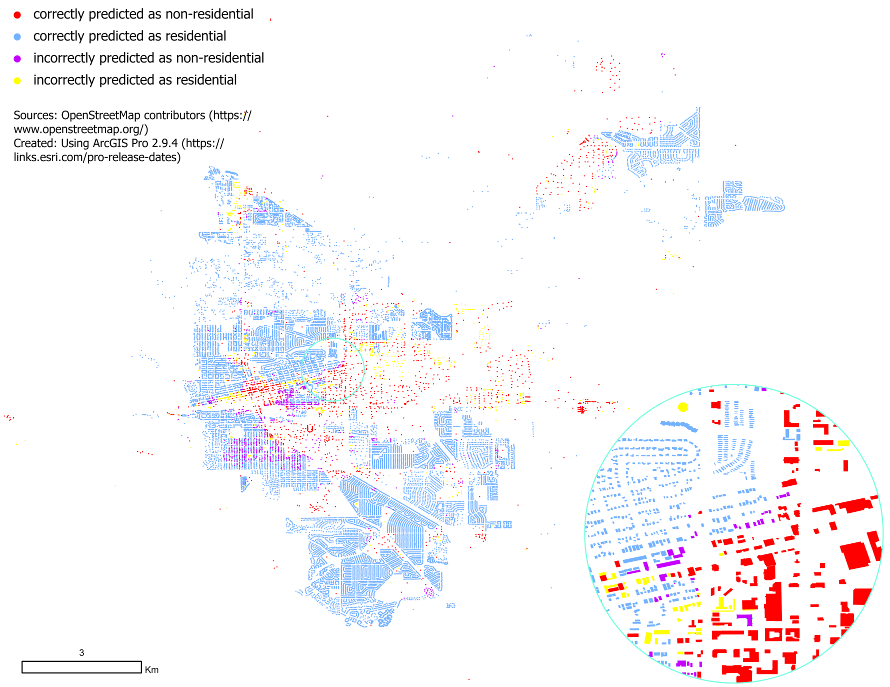

Figure 8: Residential and non-residential building type based on transfer learning model for Fairfax County transferred to City of Boulder.

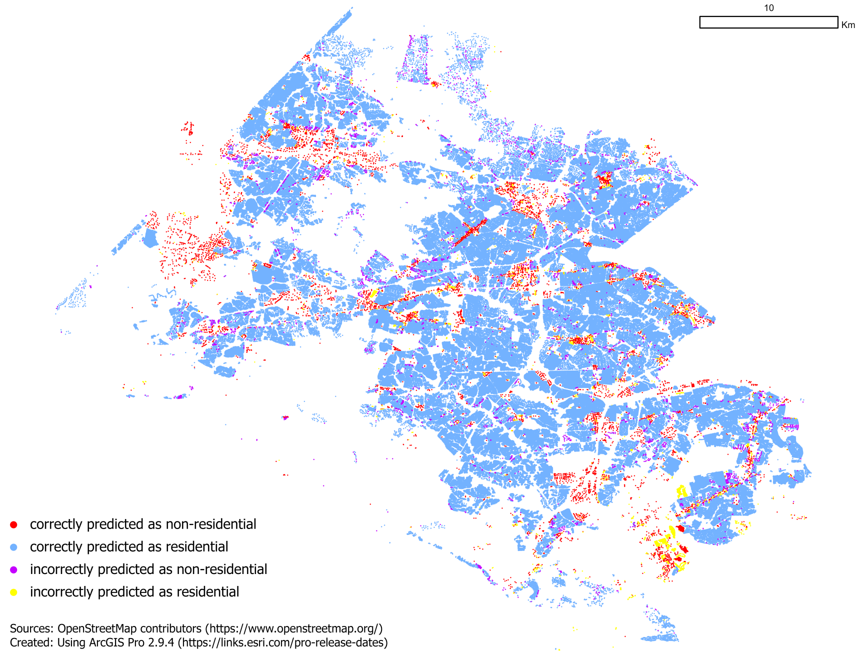

Figure 9: Residential and non-residential building type based on transfer learning model for Mecklenburg County transferred to Fairfax County.

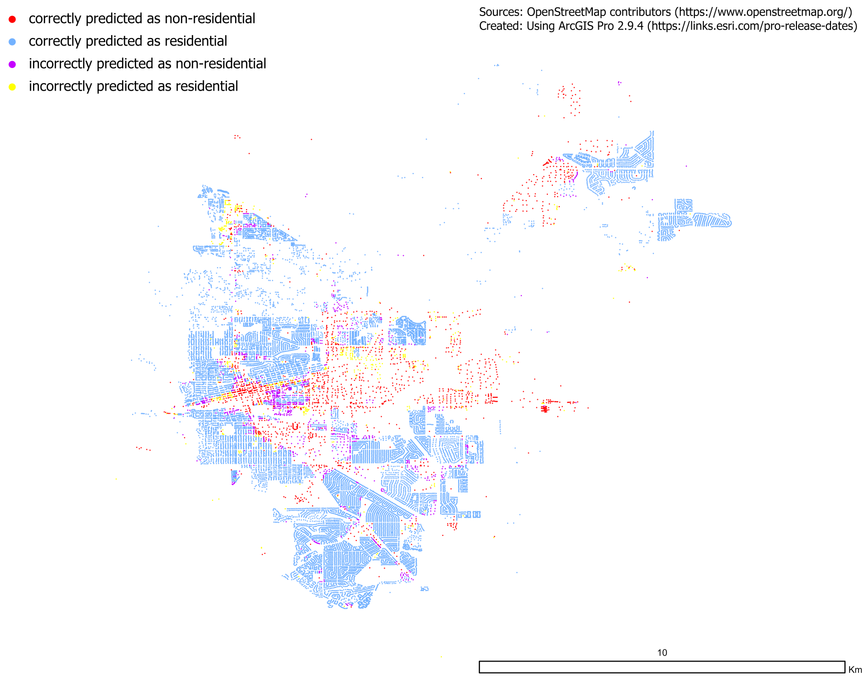

Figure 10: Residential and non-residential building type based on transfer learning model for Mecklenburg County transferred to City of Boulder.

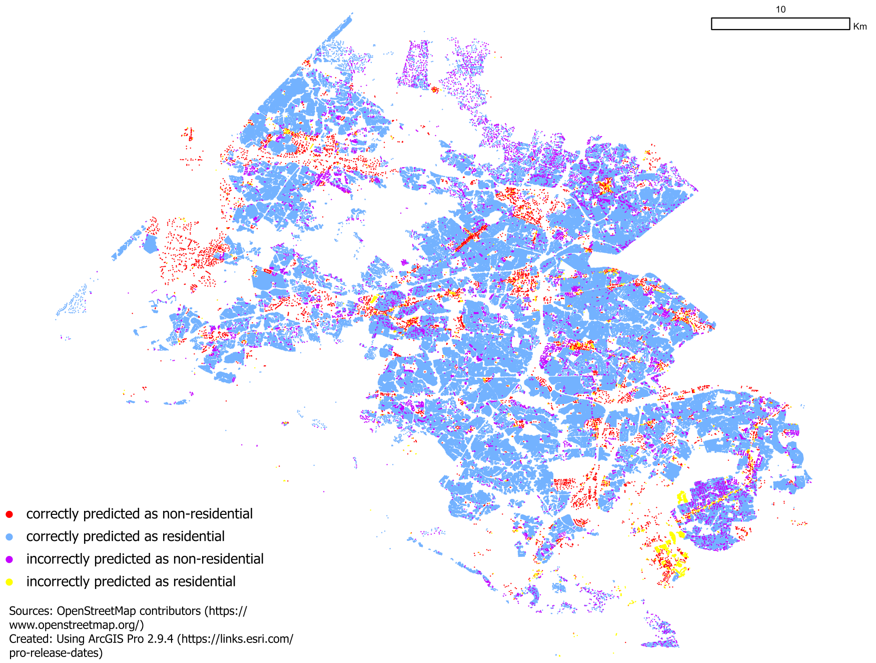

Figure 11: Residential and non-residential building type based on transfer learning model for City of Boulder transferred to Fairfax County.

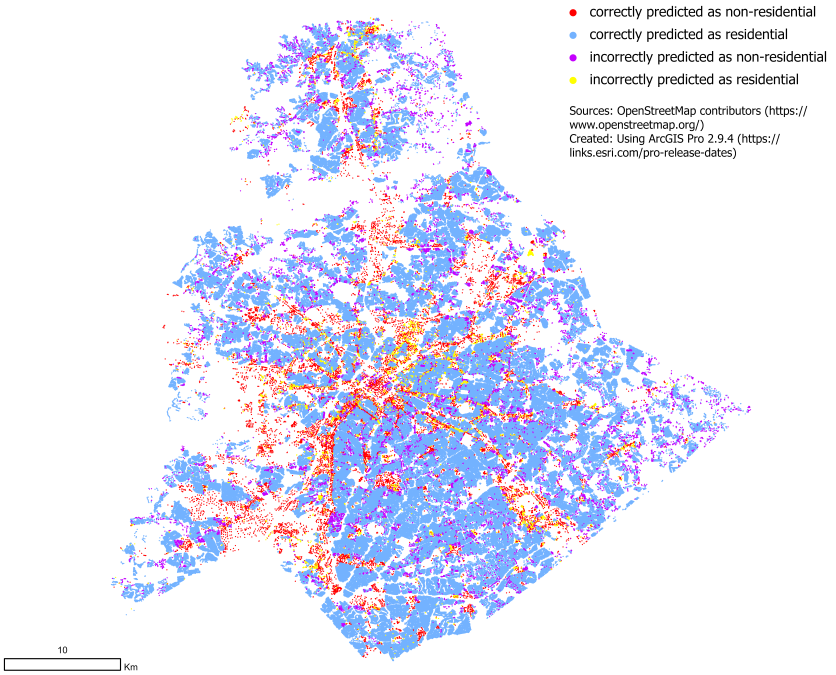

Figure 12: Residential and non-residential building type based on transfer learning model for City of Boulder transferred to Mecklenburg County.
